# The Critical Role of Steroid Regimen for Lung Repair in Experimental Diffuse Alveolar Damage

**DOI:** 10.3390/ijms27031199

**Published:** 2026-01-25

**Authors:** Aleksandr Chernov, Georgii Telegin, Evgeny Sinitsyn, Alexey Dmitriev, Viktor Palikov, Vitaly Kazakov, Maksim Rodionov, Igor Rybalkin, Tatiana Vlasik, Alexey Belogurov, Kirill Zykov

**Affiliations:** 1 Shemyakin–Ovchinnikov Institute of Bioorganic Chemistry, Russian Academy of Sciences, Moscow 117997, Russia; alexandrchernov1984@gmail.com (A.C.); belogurov@mx.ibch.ru (A.B.); 2 Branch of Shemyakin–Ovchinnikov Institute of Bioorganic Chemistry, Russian Academy of Sciences, Pushchino 142290, Russia; telegin@bibch.ru (G.T.); dmitr.lexarusbear@gmail.com (A.D.); vpalikov@bibch.ru (V.P.); vitalij.tomsk@list.ru (V.K.); 3 “Pulmonology Scientific Research Institute” Under Federal Medical and Biological Agency of Russian Federation, Moscow 115682, Russia; sinymlad@list.ru; 4 Department of Faculty Therapy and Occupational Diseases, N.A. Semashko Scientific and Educational Institute of Clinical Medicine, “Russian University of Medicine” Under the Ministry of Health of Russia, Moscow 127006, Russia; 5 “State Research Center for Applied Microbiology and Biotechnology”, Obolensk 142279, Russia; 6 A.F. Tsyb Medical Radiological Research Center, Branch of the National Medical Research Center of Radiology, Obninsk 249036, Russia; mvrodionov@inbox.ru; 7 National Medical Research Center of Cardiology Named After Academician E.I. Chazov, Ministry of Health of Russia, Moscow 121552, Russia; irybalkin@gmail.com (I.R.); tanya.vlasik@gmail.com (T.V.)

**Keywords:** glucocorticosteroids, dosing, durations, ARDS/DAD, animal models

## Abstract

Acute respiratory distress syndrome (ARDS) is a common condition among intensive care unit patients and is associated with high mortality. Currently, there are no unified therapeutic strategies, including for the use of systemic glucocorticosteroid (GCS) therapy, in the management of ARDS of various etiologies. Using our previously developed non-surgical and reproducible model of unilateral total diffuse alveolar damage (ARDS/DAD) in the left lung of ICR mice, we investigated the effects of GCS with different durations of action and administration regimens on lung function recovery. Our data show that repeated-course administration of dexamethasone promoted complete normalization of respiratory function, as well as restoration of aeration and perfusion of the left lung in mice following ARDS/DAD induction. In contrast, a single administration of the same drug or the use of a prolonged-release formulation, despite exhibiting anti-inflammatory effects, did not provide adequate lung tissue recovery and, in some cases, even exacerbated injury. These results underscore that in ARDS therapy, not just the use but the specific dosing regimen of glucocorticoids is critically important for driving complete functional and structural lung repair.

## 1. Introduction

Acute respiratory distress syndrome (ARDS) is an acute, diffuse, inflammatory injury of the lungs triggered by a predisposing risk factor such as pneumonia, extrapulmonary infection, trauma, transfusion, burn injury, aspiration, or shock. The resulting injury increases the permeability of pulmonary vessels and the alveolar epithelium, leading to pulmonary edema and gravity-dependent atelectasis, which together result in a loss of aerated lung tissue. Clinically, ARDS is characterized by arterial hypoxemia and diffuse radiographic opacities associated with shunt formation, increased alveolar dead space, and decreased lung compliance. The clinical picture depends heavily on supportive treatment measures—such as patient positioning, sedation, neuromuscular blockade, positive end-expiratory pressure, and fluid balance. Histopathological features vary and may include intra-alveolar edema, inflammation, hyaline membrane formation, and alveolar hemorrhage [1].

According to a multicenter observational study conducted in 2016 across 50 countries, the incidence of ARDS among patients admitted to intensive care units was 10.4%, with a hospital mortality rate of 40.0% [2]. Many authors emphasize that ARDS remains under-recognized and undertreated and is associated with persistently high mortality [3]. These findings underscore the urgent need to develop new therapeutic approaches to ARDS.

The most common causes of ARDS include infectious agents leading to pneumonia (59.4%) and extrapulmonary sepsis (16%) [2]. ARDS associated with SARS-CoV-2 infection has been at the center of research efforts for the past five years, given the virus’s role in more than 7.1 million deaths worldwide [4].

To date, no universally effective treatment regimens for ARDS have been developed. Current therapeutic approaches are complex and highly individualized, aimed at maintaining adequate gas exchange while minimizing ventilator-induced lung injury and addressing the underlying cause of the syndrome. Treatment generally includes the following non-pharmacological and pharmacological strategies:

1. Conservative oxygen therapy that avoids hyperoxia;
2. Prone positioning to improve ventilation–perfusion relationships;
3. Lung-protective mechanical ventilation, with early mobilization and systematic assessment of readiness for spontaneous breathing and extubation;
4. Extracorporeal membrane oxygenation (ECMO) for refractory hypoxemia; in some cases, extracorporeal carbon dioxide removal (ECCO_2_R) is used;
5. Treatment of the underlying cause—including aggressive antibacterial or antiviral therapy for sepsis or pneumonia, surgical sanitation of infectious foci, aspiration management, etc.;
6. Conservative fluid management with careful hemodynamic control to prevent iatrogenic pulmonary edema;
7. Pharmacotherapy with limited indications—systemic glucocorticosteroid (GCS) in low-to-moderate doses (particularly in COVID-19-associated ARDS, early fibroproliferative ARDS, or refractory septic shock); inhaled vasodilators (nitric oxide, prostacyclin) for short-term oxygenation improvement in refractory cases; and short courses of neuromuscular blockers (cisatracurium) in severe ARDS [5].

The role of glucocorticoids in ARDS remains a matter of active debate, with no consensus on their precise indications, optimal dose, or duration.

A meta-analysis of 20 randomized controlled trials (*n* = 3890) assessing GCS efficacy in ARDS showed that GCS use was associated with reduced mortality (OR = 0.80, 95% CI: 0.71–0.91, *p* = 0.001). However, observational studies linked GCS use to higher mortality (OR = 1.16, 95% CI: 1.04–1.29, *p* = 0.001). Factors influencing treatment efficacy included dosage, GCS type, and ARDS etiology [6].

Notably, dexamethasone was the first treatment shown to reduce mortality in patients with COVID-19-related lung injury receiving mechanical ventilation. However, among patients not requiring respiratory support, dexamethasone use tended to increase mortality [7]. Subsequent studies demonstrated that increasing the dose to 20 mg/day did not further reduce mortality in ventilated patients [8,9]. A meta-analysis of nine RCTs (*n* = 2740) found no significant difference between glucocorticoid doses in terms of 28-day mortality, hospital stay, or adverse events in COVID-19 patients [10].

Thus, there are still no standardized therapeutic strategies for glucocorticoid use in ARDS of various etiologies. Clinical and preclinical data are insufficient in determining whether a short, high-dose pulse or a prolonged, moderate-dose course—or a strategy of repeated administration—is more effective in promoting the restoration of alveolar structure and function. This gap in knowledge significantly hinders the development of standardized therapeutic protocols. Determining the optimal treatment strategy requires a mechanistic rationale supported by experimental data. In this regard, animal models of diffuse alveolar damage (DAD)—the histopathological hallmark of ARDS—are invaluable.

Previously, we developed and described a non-lethal unilateral mouse model of ARDS/DAD induced by intrabronchial administration of lipopolysaccharide and α-galactosylceramide [11]. This model allows for the evaluation of both the injury and recovery phases of lung structure and function, including under various therapeutic interventions [12,13,14]. The present study was designed to directly investigate how the glucocorticoid regimen—specifically, the duration of drug action and the frequency of administration—influences functional and structural recovery from established DAD. Using our validated murine model, we compared the effects of a long-acting GCS (dexamethasone) administered either as a single dose or as a repeated course against a prolonged-release formulation. We hypothesized that the regimen critically determines therapeutic efficacy, with repeated administration being necessary to sustain the biological signals required for complete alveolar repair. Our findings provide novel experimental evidence that shifts the paradigm from simply considering steroid use to strategically optimizing their dosing schedule for lung recovery.

## 2. Results

### 2.1. Respiratory Function Assessment

Measurement of respiratory parameters in mice with ARDS/DAD treated with GCS is presented in Table 1. We observed the development of respiratory dysfunction associated with ARDS/DAD, characterized by decreased tidal volume and maximal expiratory flow, while respiratory rate was significantly increased on days 7, 14, and 30 in all mice with experimental ARDS/DAD (Table 1). A single administration of dexamethasone and betamethasone showed no therapeutic effect, as respiratory function parameters were statistically no different and remained at the same level as in animals receiving saline. In contrast, repeated administration of dexamethasone demonstrated a clear therapeutic benefit, with improvement observed as early as day 14. The respiratory rate, tidal volume, and maximal expiratory flow values in this group approached those of intact animals and became fully comparable by day 45 (Table 1).

### 2.2. Lung Computed Tomography

Three-dimensional CT images of lung volume and mean density were obtained using a computed tomography scanner on days 7, 14, 30, and 45 after ARDS/DAD induction. Evaluation of mean lung density (in Hounsfield units) and total lung volume (in mm^3^) provided important information on the functional state of the lungs and the dynamics of post-ARDS/DAD recovery under different drug treatments (Table 2).

In Groups 3 and 5, a slight decrease in lung density relative to the control group was observed at early time points (day 7). However, from day 14 onward, the recovery of normal lung structure in these groups proceeded considerably more slowly. In contrast, Group 4 demonstrated a rapid restoration of lung aeration, which was essentially complete by day 30.

**Figure 1:**
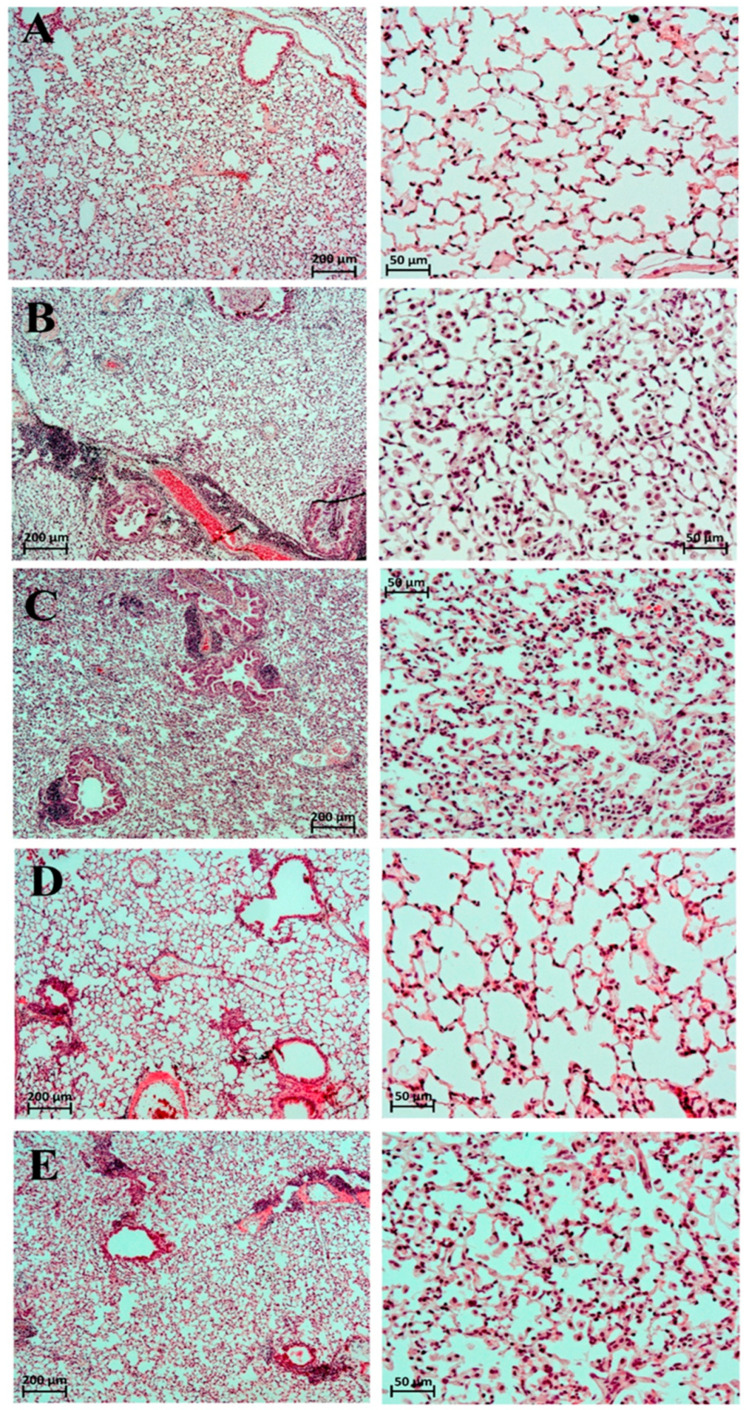
Fragments of the left lung lobe in male ICR mice 45 days after induction of inflammation in the ARDS/DAD model following single and repeated administration of dexamethasone, saline, and betamethasone. Semi-quantitative scoring of peribronchial and perivascular mononuclear infiltration and mononuclear infiltration of alveolar walls and lumen. Hematoxylin and eosin staining. Magnification ×50 (left column) and ×200 (right column). (**A**) Intact animals: PB infiltration = 0, PV infiltration = 0, alveolar wall/lumen infiltration = 0; (**B**) induction of ARDS/DAD with single treatment by saline: PB = 2, PV = 3, AW/AL = 3; (**C**) induction of ARDS/DAD with single treatment by dexamethasone: PB = 2, PV = 3, AW/AL = 3; (**D**) induction of ARDS/DAD with repeated treatment by dexamethasone: PB = 1, PV = 2, AW/AL = 1; (**E**) induction of ARDS/DAD with single treatment by betamethasone: PB = 1, PV = 2, AW/AL = 2.

Histological analysis of the lungs in ARDS/DAD was characterized by infiltration of mononuclear cells in the perivascular (PV) and peribronchial (PB) spaces, in the alveolar wall (AW), and in the alveolar lumen (AL) (Figure 1, Table 3). In the lungs of intact male ICR mice, no deviations from the norm were detected—all evaluated parameters scored 0 points (Table 3).

In the ARDS/DAD model with saline administration, by day 45, residual signs of total lesion of the left lung lobe were noted in all cases, with a significant reduction in its volume and total hypoventilation (average macroscopic score = 3.50; histological hypoventilation score = 2.43). Moderate focal perivascular (average = 2.86) and mild focal peribronchial (average = 2.14) mononuclear infiltration were commonly observed (Table 3, Figure 1).

**Figure 2:**
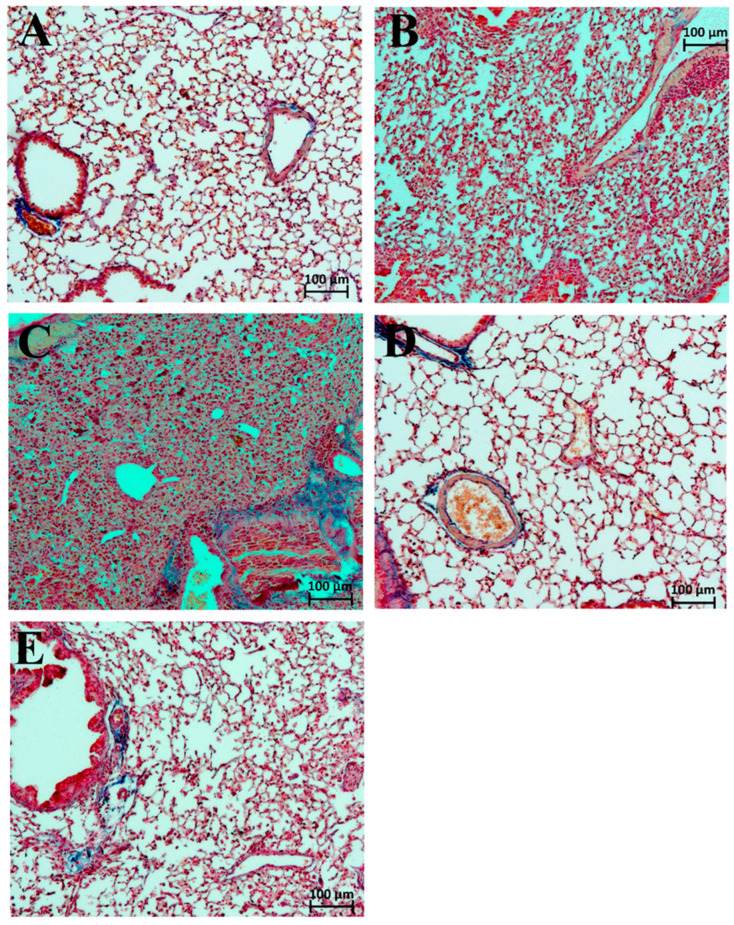
Fragments of the left lung lobe of male ICR mice on day 45 in the DAD model after single and repeated administration of dexamethasone, saline, and betamethasone. Semi-quantitative scoring of fibrosis (perivascular and peribronchial fibrosis, thickening and fibrotic changes of interalveolar septa; 0–5 points). Mallory staining. Magnification ×100. (**A**) Intact animals: fibrosis = 0; (**B**) induction of ARDS/DAD with single treatment by saline: fibrosis = 2; (**C**) induction of ARDS/DAD with single treatment by dexamethasone: fibrosis = 3; (**D**) induction of ARDS/DAD with repeated treatment by dexamethasone: fibrosis = 1; (**E**) induction of ARDS/DAD with single treatment by betamethasone: fibrosis = 2.

The alveolar walls and lumens were also diffusely infiltrated with numerous mononuclear cells (2.86 points). The lumen of many bronchi was partially or completely obstructed. In one animal, small necrotic foci in the regeneration stage were detected in the left lung lobe (average score = 0.29). In the perivascular and peribronchial spaces, broad areas of loose fibrous connective tissue were observed; in collapsed lung regions, interalveolar septa were thickened and fibrotically altered (Figure 2). The average degree of fibrosis in this group was 2.00 points, and the Kernohan index was 0.51 ± 0.08 (vs. 0.35 ± 0.01 in intact animals).

After a single intravenous administration of dexamethasone, no significant positive trends were observed (Table 3, Figure 1). On the contrary, mononuclear infiltration of the alveolar walls and lumens, as well as hypoventilation signs, worsened compared to saline (averaging 3.00 and 2.46 points, respectively). This was confirmed by the macroscopic score of the left lung lobe—all animals receiving dexamethasone showed maximal lesions (5.00 points). Fibrotic changes in the left lobe also trended negatively (2.80 points, Figure 2). The Kernohan index was comparable to the saline group (0.49 ± 0.02) (Table 4).

A single administration of betamethasone by day 45 of observation contributed to reduced perivascular (2.00) and peribronchial (1.80) mononuclear infiltration, as well as infiltration of alveolar walls and lumen (2.00) (Table 3, Figure 1). However, betamethasone administration did not result in significant improvement in ventilation of the affected lobe (areas of collapse scored 2.20), which was reflected in the macroscopic score—all animals exhibited total damage of the left lung lobe (5.00). The degree of fibrosis (2.00) and the Kernohan index (0.50 ± 0.04) did not differ from those in the saline group (Figure 2, Table 4).

In contrast, repeated administration of dexamethasone to male ICR mice with ARDS/DAD resulted in marked improvement of the morphologic picture in the left lung lobe by day 45 across all key parameters. The average score of mononuclear infiltration of the alveolar walls and lumen was 1.50 points, while scores of peribronchial and perivascular infiltration were 1.25 and 2.00 points, respectively (Table 3, Figure 1). Furthermore, the left lung lobe in this group was more actively involved in gas exchange—areas of atelectasis and fibrosis averaged 1.25 points (macroscopic score = 1.00), and the Kernohan index did not significantly differ from control values (0.41 ± 0.08) (Figure 2, Table 4).

The diameter of cardiomyocytes in both ventricles in DAD mice exceeded that of intact animals, generally showing a direct proportional relationship between the severity of myocardial hypertrophy, the Kernohan index, and the extent of lung pathology (Table 4). The largest cardiomyocyte diameters were observed in the betamethasone group (13.4 ± 0.4 µm), and the smallest in the repeated-dexamethasone group.

Thus, repeated intravenous dexamethasone administration demonstrated high efficacy in the ARDS/DAD mouse model, leading to early and sustained re-engagement of the affected lobe in systemic gas exchange. This was supported by low fibrosis scores of the left lung lobe and favorable Kernohan index values, which reflect pulmonary vascular capacity and, consequently, systemic circulatory load. Conversely, single-dose dexamethasone had a negative impact on inflammatory outcomes. Administration of a prolonged-release GCS formulation improved the general inflammatory picture but failed to ensure effective reintegration of the affected lobe into systemic gas exchange, resulting in increased right-ventricular load.

## 3. Discussion

This study presents a comprehensive comparative analysis of the efficacy of various GCS therapy regimens in an experimental model of ARDS/DAD induced by a combination of LPS and α-galactosylceramide.

The pathophysiology of ARDS involves a complex cascade of inflammatory mediators that rapidly and severely damage the lungs. This process disrupts both the alveolar epithelium and vascular endothelium, leading to diffuse alveolar injury. The initial exudative phase is characterized by protein-rich pulmonary edema resulting from increased vascular permeability and cellular necrosis. This phase is followed by a fibroproliferative phase, marked by interstitial and intraluminal fibrosis and proliferation of type II alveolar epithelial cells [15]. Given the complexity of these mechanisms, a wide range of pharmacological treatments have been proposed.

Despite ARDS being recognized as a distinct clinical entity for more than half a century, no universal therapies or approaches have emerged to significantly improve survival. The heterogeneity of ARDS—in terms of etiology, clinical manifestations, and response to treatment—complicates the search for optimal management strategies. The use of GCSs has been considered a potential treatment strategy for ARDS due to their ability to reduce systemic inflammatory responses [6].

GCSs are thought to exert their effects in ARDS through at least three mechanisms. First, they may supplement endogenous glucocorticoid activity and mitigate hypothalamic–pituitary–adrenal axis dysfunction. Second, GCSs attenuate cytokine release, thereby reducing endothelial and alveolar epithelial injury. They modulate pro-inflammatory transcription factors such as nuclear factor κB and suppress the production of pro-inflammatory cytokines including TNF-α, IL-1, IL-6, and IL-8 [16,17]. Inhibition of IL-8, a potent neutrophil chemoattractant, decreases the recruitment of neutrophils and macrophages into the alveoli. Reduction in pro-inflammatory mediators and leukocyte infiltration may, in turn, decrease alveolar fluid accumulation, improving gas exchange and lung compliance. Finally, GCS may promote clearance of alveolar edema by accelerating the resolution of epithelial injury [18].

However, the wide range of side effects limits the broad clinical use of systemic glucocorticoids. The risks associated with GCS therapy are known to depend on both dose and duration. Generally, low-dose, short-term GCS therapy does not cause significant adverse effects. Prolonged use, however, may result in undesirable outcomes such as weight gain, hyperglycemia, diabetes, osteoporosis, glaucoma, cataracts, and psychiatric disorders. Drug interactions between GCSs and anticoagulants, antidiabetic agents, diuretics, or antihypertensive drugs may alter their therapeutic potential, rendering treatment ineffective or increasing the frequency of side effects [19].

Another unresolved issue concerns the optimal regimen for GCS administration in ARDS, as available clinical data remain contradictory. A large meta-analysis of 11 observational studies and 9 RCTs involving 3890 patients showed that the effectiveness of GCS in reducing ARDS mortality varied depending on the type and dosage of GCS as well as the underlying etiology. Current evidence does not support the routine use of GCS in ARDS, as protective effects were observed in RCTs but increased mortality was reported in observational studies [6].

Previous findings demonstrated that dexamethasone at a dose of 6 mg per day for ten days reduced mortality risk in hospitalized COVID-19 patients receiving mechanical ventilation, whereas in patients without respiratory failure, there was a 19% increase in mortality [7]. Moreover, no evidence was found that higher doses further decreased mortality risk in these patients [9].

Data regarding the duration of GCS administration also vary. In one RCT (*n* = 1294), prolonged dexamethasone treatment was associated with significantly increased in-hospital mortality in patients with severe COVID-19 [20]. Conversely, a meta-analysis of 43 RCTs found that longer (≥7 days) GCS therapy, as well as early initiation (≤72 h) and low doses, were associated with reduced short-term mortality in severe community-acquired pneumonia or ARDS [21].

In our study, we also observed differences in the effects of short-term and long-term dexamethasone administration, which may stem from fundamental differences in the mechanisms of GCS action depending on the duration of exposure. This applies not only to the severity of the anti-inflammatory effect but also its impact on different phases of the pathological process (acute inflammation vs. repair and fibrosis).

A single administration may provide sufficient concentration for a potent but transient transrepression (NF-κB inhibition), temporarily reducing inflammatory activity. However, this stimulus is insufficient in initiating long-term anti-inflammatory effects and regulating reparative processes that require transactivation (activation of specific gene transcription following glucocorticoid receptor binding to glucocorticoid response elements) [22].

Repeated administration maintains a steady drug concentration, allowing continuous suppression of pro-inflammatory signals and modulation of gene expression through transactivation. Theoretically, this may lead to activation of genes encoding antiproteases, induction of apoptosis in activated inflammatory cells (neutrophils, lymphocytes), and stimulation of proteins protecting epithelial and endothelial cells [23].

Conversely, single-dose administration may induce a “rebound” effect: abrupt suppression of inflammation may impair natural clearance of apoptotic cells and initiation of repair processes, resulting—as observed in our study—in enhanced fibrosis and atelectasis development [24]. Repeated dosing likely enhances reparative processes while suppressing excessive fibroblast proliferation and collagen synthesis.

The paradoxical worsening of histological outcomes after single-dose dexamethasone, despite some functional improvement, can be explained by an imbalance between transient inflammation suppression and long-term disruption of repair. A single high dose likely induces strong transrepression of inflammatory mediators, temporarily improving respiratory function, but fails to modulate key resolution processes such as neutrophil apoptosis or macrophage polarization. Abrupt cessation of anti-inflammatory signaling may lead to a “rebound” inflammatory response and dysregulated fibrosis.

Clinically, this was reflected by worsening morphological outcomes in Group 3 compared with the placebo group (saline). Despite a modest trend toward functional improvement by day 45, histological analysis revealed intensified alveolar infiltration, atelectasis, and fibrosis. Macroscopic examination showed total involvement of the left lung lobe in all animals. This suggests that single-dose dexamethasone pulse therapy may trigger an inadequate or incomplete inflammatory response that temporarily suppresses symptoms but ultimately leads to more severe tissue injury and impaired repair [25,26]. This phenomenon warrants special attention in clinical practice.

In contrast, prolonged dexamethasone therapy maintained consistent suppression of pro-inflammatory signals and possibly activated anti-inflammatory gene expression via transactivation mechanisms, thereby promoting organized inflammation resolution and preventing excessive fibrosis.

In our study, Group 4 (multiple dexamethasone doses) was the only group demonstrating complete normalization of respiratory function (spirometry), restoration of left lung aeration to near-normal levels (CT data), and significant improvement in histopathological parameters by days 30–45. Reduction in the Kernohan index and absence of marked right ventricular hypertrophy indicated pulmonary vascular remodeling and decreased pulmonary arterial pressure [27]. This reflects not only suppression of inflammation but also prevention of long-term DAD complications such as pulmonary hypertension and fibrosis. Consequently, our data offer a direct mechanistic explanation for clinical observations where a longer course of dexamethasone proved beneficial. They also highlight the possibly risk inherent in single-dose or short-pulse strategies, which our model shows can transiently suppress inflammation without supporting the sustained biological processes necessary for functional and structural recovery.

Consistent with our findings, other authors concluded that the beneficial effects of GCS in severe COVID-19 infection depend on treatment duration: GCS therapy should last at least 10 days, as low-dose dexamethasone for 10 days outperformed 3-day methylprednisolone pulses in reducing in-hospital mortality and the need for ICU admission or mechanical ventilation in noncritical COVID-19 patients [28].

The prolonged-release betamethasone used in Group 5 effectively suppressed perivascular and peribronchial inflammation; however, this did not translate into functional improvement. Lung volume and aeration did not recover, extensive atelectasis persisted, and the highest degree of right ventricular hypertrophy was observed. This likely indicates that sustained systemic GCS exposure, while controlling inflammation, may impair active repair and lung tissue regeneration [29,30], possibly by inhibiting type II pneumocyte proliferation and angiogenesis. Consequently, hypoventilation and increased right heart load persist.

Dexamethasone administered as a course creates fluctuating peaks and troughs in drug concentration. After each dose, therapeutic levels are reached and then decline, allowing partial physiological recovery. This pulsatile concentration pattern may be more physiological, providing “rest” periods for activation of intrinsic repair mechanisms. In contrast, betamethasone produces a continuous, plateau-like concentration for many days or weeks. Such sustained glucocorticoid signaling completely suppresses the immune system, preventing adaptive recovery. Continuous inhibition of angiogenesis by betamethasone also impedes microcirculatory restoration, as reflected by the high Kernohan index and right ventricular hypertrophy (signs of pulmonary hypertension).

Our data show that fibrosis in the betamethasone group was equivalent to that of the placebo group (2.00 points), whereas dexamethasone therapy significantly reduced it (1.25 points). This may be due to prolonged GCS exposure disrupting the balance between collagen synthesis and degradation, leading to scarring rather than regeneration.

A limitation of this study is the lack of molecular data clarifying the mechanisms underlying observed differences between treatment regimens (e.g., TGF-β, NF-κB, apoptosis, and proliferation markers). Elucidating these mechanisms is a critical next step in further research. Another limitation is the experimental nature of this model, which requires caution when extrapolating findings to humans and necessitates additional clinical studies.

Patient age also plays an important role in GCS therapy outcomes. In severe COVID-19 patients on mechanical ventilation, short dexamethasone courses significantly reduced mortality only in patients under 65 years (by 53%), whereas no benefit was observed in older individuals. This finding highlights the need to account for patient phenotypic characteristics in future clinical research [31].

Based on our results, future studies should aim to determine the optimal timing and duration of dexamethasone therapy, elucidate molecular and cellular mechanisms underlying the paradoxical worsening of histological outcomes after single dosing, and explore combination regimens of dexamethasone with pro-reparative agents (e.g., growth factors) to enhance tissue recovery.

## 4. Methods and Materials

### 4.1. Animals

This study used 35 male ICR mice with an average body weight of 34.2 ± 1.4 g. The animals were housed under standard conditions at the Laboratory Animal Breeding Facility of the Branch of the Shemyakin–Ovchinnikov Institute of Bioorganic Chemistry, Russian Academy of Sciences (the Unique Research Unit “Bio-Model of the BIBCh RAS”; Bioresource Collection of SPF Laboratory Rodents for Fundamental, Biomedical, and Pharmacological Studies, contract 075-15-2025-486).

All experiments and manipulations were approved by the Institutional Animal Care and Use Committee (IACUC No. 926/22, dated 11 April 2022).

### 4.2. Model of Diffuse Alveolar Damage

ARDS/DAD was induced by intrabronchial administration of 200 μL of a mixture containing 100 μL of lipopolysaccharide (LPS) from *Salmonella enterica* (1 mg/mL, MilliporeSigma, Burlington, MA, USA) and 100 μL of α-galactosylceramide (50 μg/mL, Avanti Polar Lipids, Alabaster, AL, USA) into the left lung, as previously described [11]. Briefly, the trachea was intubated with an intravenous 20G catheter that was put forward into the left mainstem bronchus, with the animal lying in the lateral position. Using an insulin syringe, the inductor mixture was quickly injected into the bronchus. As premedication during the intubation, a short-acting hypnotic agent Propofol was used (Hana Pharmaceutical, Co., Ltd., Seoul, Republic of Korea). The hypnotic agent was administered as an intravenous bolus into the lateral tail vein at a dose of 20 mg/kg.

### 4.3. Experimental Groups

Mice were randomly divided into five groups with seven animals in each group. A sample size of 7 provides sufficient power for statistical tests to detect meaningful differences, preventing Type II errors (missing a real effect), and ensure they meet ethical standards of 3R.

Description of experimental groups:

Group 1 (control): Intact animals without ARDS/DAD induction.

Group 2 (DAD + saline, negative control): Induction of ARDS/DAD followed by a single intravenous (i.v.) injection of 100 µL of 0.9% saline.

Group 3 (DAD + single dexamethasone): Induction of ARDS/DAD followed by a single i.v. injection of dexamethasone (0.5 mg/kg).

Group 4 (DAD + repeated dexamethasone course): Induction of ARDS/DAD followed by repeated i.v. injections of dexamethasone (1 mg/kg, ten injections every three days over 28 days).

Group 5 (DAD + betamethasone, single administration): Induction of ARDS/DAD followed by a single i.v. injection of betamethasone (0.875 mg/kg).

Animals were monitored for 45 days after induction daily. After the completion of the experiment, all animals were euthanized.

### 4.4. Spirometry

Respiratory parameters were assessed using a computerized PowerLab 8/35 system equipped with a spirometer module and a breathing head. The animal was placed in a mouse restrainer (holding device) and allowed at least 2 min to calm down and assume a comfortable body position. A breathing mask was gently applied to the animal’s nose, and recording was initiated in the LabChart software v.8 for 10 s. The following parameters were measured: respiratory rate (breaths per minute); maximal expiratory flow, mL/s (MEF); and tidal volume, mL (TV).

Descriptive statistics were applied to all quantitative data: the mean value and standard deviation were calculated and, along with the N value (number of variants per group), are presented in the tables.

### 4.5. Lung Computed Tomography (CT)

To assess the condition of the lungs, an MRS*CT/PET scanner (MR Solution, Surey, UK) was used. Scanning parameters: energy, 40 kVp; exposure, 100 ms; current, 1 mA; and rotation step, 1°. During scanning, animals were anesthetized with 3% isoflurane and placed in a specialized bed maintained at 37 °C. The obtained CT images were processed using VivoQuant v.4.0 software (Invicro, London, UK). The volume and mean tissue density of the left (experimental) and right (control) lungs were measured after segmentation of these lobes in automatic mode (using a density threshold of −300 to −800 Hounsfield units) or manually. The volume is expressed in mm^3^, and density in Hounsfield units (HU ± SEM).

### 4.6. Histology

The heart and lungs, fixed as an organ complex in 10% neutral buffered formalin, were subsequently dissected: tissue fragments were taken from each lung lobe (one from the left lobe, four from the right lobes). Serial cross-sections of the heart were prepared to allow detailed examination of the myocardium of the left and right ventricles. Tissue samples were rinsed in running water, dehydrated through a graded series of ethanol, and embedded in paraffin. Paraffin sections 4–5 µm thick were stained with hematoxylin and eosin and by the Mallory method. Slides were examined by standard light microscopy using an AxioScope.A1 microscope (Carl Zeiss, Oberkochen, Germany). Microphotographs of histological specimens were obtained using a high-resolution Axiocam 305 color camera (Carl Zeiss, Germany) and processed with ZEN 2.6 lite software (Carl Zeiss, Germany).

During histological analysis of the lungs, the following morphological features were evaluated: peribronchial and perivascular mononuclear infiltration; mononuclear infiltration of the alveolar walls and lumens; areas of lung tissue collapse; presence or absence of necrotic foci; and the degree of fibrosis. The severity of various inflammatory changes in the lungs and the degree of pneumofibrosis were evaluated semi-quantitatively (scored) according to the following scale: 0—within normal limits; 1—minimal; 2—mild; 3—moderate; 4—marked (noticeable changes with potential for further progression); 5—severe (maximal, representing total lobe damage). Macroscopic changes in the left lung lobe observed during necropsy were evaluated using a 5-point scale: 0—within normal limits; 1—localized root or apical lesion; 2—large focal cranial lesion; 3—large focal median lesion; 4—subtotal lesion (except for small, usually caudal, unaffected areas); 5—total lesion of the left lobe.

Using ZEN 2.6 lite software (Carl Zeiss, Germany), the Kernohan index (ratio of vascular wall thickness to vessel lumen radius) was assessed in the blood vessels of the left lung lobe as an important indicator of the microcirculatory capacity of the pulmonary circulation. In the heart, special attention was paid to the condition of the myocardium of the right ventricle as a potential marker of pulmonary hypertension resulting from total or subtotal atelectasis of the left lung lobe, and to the presence of fibrotic foci in the myocardium of both ventricles. Quantitative assessment of cardiomyocyte diameter in the left and right ventricles was performed on longitudinal sections of myocardial fibers (at least 100 heart cells in a minimum of 10 microscopic fields).

### 4.7. Statistical Analysis

The results are presented as mean ± SEM. Statistical analysis of the obtained data was performed using Statistica software (StatSoft^®^, v.12.6, Tulsa, OK, USA). Quantitative data from functional tests were analyzed using the Mann–Whitney test for pairwise group comparisons. Statistical analysis was conducted with Statistica 7.1 software. Differences were considered significant at a 5% significance level.

## 5. Conclusions

The obtained data suggest that when investigating various treatment approaches for ARDS/DAD in models simulating COVID-19-related lung injury, it is essential to consider the time interval between the injurious exposure and drug administration, as well as to monitor not only the acute phase but also the convalescent period. This is feasible when using non-lethal models, one of which was employed in the present study. Our findings clearly demonstrate that in the murine ARDS/DAD model, only repeated-course administration of dexamethasone results in sustained functional and morphological recovery of the lungs, preventing the development of fibrosis and pulmonary hypertension. In contrast, a single dose of the same drug or the use of a prolonged-release formulation, despite exhibiting anti-inflammatory activity, fails to ensure adequate restoration of lung tissue and may even exacerbate injury. These results underscore that in ARDS therapy, not just the use but the specific dosing regimen of glucocorticoids is critically important for driving complete functional and structural lung repair.

## Figures and Tables

**Table 1:** Results of spirometric assessment of lung function in mice with ARDS/DAD (*n* = 7 per group).

Testing Day	Respiratory Rate, Breaths/min	Tidal Volume, mL	Maximal Expiratory Flow, mL/s
	Mean ± SD	Mean ± SD	Mean ± SD
Group 1. Intact, without ARDS/DAD induction			
Day 7	195 ± 9.1	0.608 ± 0.020	7.32 ± 0.17
Day 14	203 ± 7.0	0.605 ± 0.016	7.20 ± 0.28
Day 30	201 ± 10.1	0.629 ± 0.013	7.22 ± 0.32
Day 45	197 ± 7.8	0.603 ± 0.020	7.40 ± 0.44
Group 2. DAD + saline, single administration			
Day 7	347 ± 15.3 *	0.260 ± 0.006 *	3.45 ± 0.20 *
Day 14	328 ± 12.9 *	0.347 ± 0.019 *	4.47 ± 0.35 *
Day 30	270 ± 26.9 *	0.449 ± 0.023 *	6.27 ± 0.07
Day 45	218 ± 12.7	0.572 ± 0.039	6.96 ± 0.13
Group 3. DAD + dexamethasone, single administration			
Day 7	338 ± 13.5 *	0.283 ± 0.030 *	3.49 ± 0.10 *
Day 14	308 ± 8.7 *	0.338 ± 0.032 *	4.01 ± 0.23 *
Day 30	247 ± 17.1 *	0.430 ± 0.029 *	6.58 ± 0.30
Day 45	212 ± 7.5	0.572 ± 0.041	7.03 ± 0.10
Group 4. DAD + dexamethasone, repeated course administration			
Day 7	311 ± 9.2 *#	0.279 ± 0.015 *	4.95 ± 0.25 *#
Day 14	265 ± 17.2 *#	0.489 ± 0.043 *#	5.91 ± 0.23 *#
Day 30	218 ± 9.7 #	0.575 ± 0.022 #	6.74 ± 0.35
Day 45	198 ± 6.3	0.652 ± 0.024	7.36 ± 0.14
Group 5. DAD + betamethasone, single administration			
Day 7	340 ± 18.1 *	0.256 ± 0.027 *	3.37 ± 0.19 *
Day 14	305 ± 13.8 *	0.352 ± 0.020 *	4.01 ± 0.12 *
Day 30	261 ± 12.9 *	0.433 ± 0.016 *	6.21 ± 0.58
Day 45	224 ± 13.1	0.552 ± 0.029	7.10 ± 0.29

* *p* ≤ 0.05 vs. Group 1 (no ARDS/DAD modeling), Mann–Whitney pairwise comparison; # *p* ≤ 0.05 vs. Group 2 (ARDS/DAD + saline), Mann–Whitney pairwise comparison.

**Table 2:** Computed tomography results for lung volume and density in mice with ARDS/DAD (*n* = 7 per group). Treatment by repeated-course dexamethasone treatment induces fast recovery of the injured lung in ICR mice with DAD.

Time (Days)	Right Lung Volume, mm^3^ (Mean)	Right Lung Density, HU (Mean)	Left Lung Volume, mm^3^ (Mean)	Left Lung Density, HU (Mean)	Left/Right Lung Density Ratio
Group 1. Intact, without ARDS/DAD induction					
7	673	−406	269	−440	1.08
14	688	−457	254	−421	0.92
30	707	−417	261	−452	1.08
45	664	−389	252	−438	1.13
Group 2. DAD + saline, single administration					
7	720	−453	259	21	0.05
14	721	−404	185	−91	0.23
30	832	−431	223	−192	0.46
45	798	−458	193	−231	0.50
Group 3. DAD + dexamethasone, single administration					
7	660	−408	242	1	0.002
14	925	−435	167	−27	0.06
30	865	−430	140	−35	0.08
45	853	−416	118	−56	0.13
Group 4. DAD + dexamethasone, repeated course administration					
7	650	−439	231	−39	0.09
14	714	−441	154	−175	0.39
30	688	−479	192	−412	0.86
45	706	−465	214	−406	0.87
Group 5. DAD + betamethasone, single administration					
7	709	−434	270	19	0.04
14	839	−416	206	−5	0.01
30	932	−471	125	−20	0.04
45	867	−422	134	−56	0.13

**Table 3:** Summary of macroscopic and microscopic evaluation scores of the left lung lobe in male ICR mice with DAD, after single and repeated administration of dexamethasone, saline, and betamethasone on day 45 of observation (*n* = 7 per group). Reduced peribronchial and perivascular mononuclear infiltration in the lung and mononuclear infiltration of the wall and lumen of the alveoli in the repeated-course dexamethasone-treated ICR mice with DAD.

Experimental Group	Macroscopic Changes in the Left Lobe During Necropsy	Peribronchial Mononuclear Infiltration	Perivascular Mononuclear Infiltration	Mononuclear Infiltration of Alveolar Walls and Lumen	Areas of Collapse	Necrosis	Degree of Fibrosis
Group 1 Intact, without ARDS/DAD ind	0	0	0	0	0	0	0
Group 2 DAD + saline, single administration	3.50 ± 0.48	2.14 ± 0.22	2.86 ± 0.43	2.86 ± 0.62	2.43 ± 0.34	0.29 ± 0.12	2.00 ± 0.63
Group 3 DAD + dexamethasone, single administration	5.00 ± 0.99	1.69 ± 0.28	2.77 ± 0.46	3.00 ± 0.30	2.46 ± 0.29	0	2.80 ± 0.45
Group 4 DAD + dexamethasone, repeated-course administration	1.00 ± 0.32	1.25 ± 0.13	2.00 ± 0.24	1.50 ± 0.44	1.25 ± 0.54	0	1.25 ± 0.19
Group 5 DAD + betamethasone, single administration	5.00 ± 0.25	1.80 ± 0.41	2.00 ± 0.37	2.00 ± 0.27	2.20 ± 0.33	0	2.00 ± 0.36

**Table 4:** Summary of morphometric parameters of vascular permeability of the left lobe (Kernohan index) and cardiomyocyte diameter in the right (RV) and left (LV) ventricles of male ICR mice with DAD after single and repeated administration of dexamethasone, saline, and betamethasone on day 45 of observation (*n* = 7 per group).

Experimental Group	Kernohan Index	Cardiomyocyte Diameter (µm)	
		RV	LV
Group 1 Intact, without ARDS/DAD ind	0.35 ± 0.01	11.5 ± 0.5	13.6 ± 0.5
Group 2 DAD + saline, single administration	0.51 ± 0.08	12.7 ± 1.3	14.4 ± 0.8
Group 3 DAD + dexamethasone, single administration	0.49 ± 0.02	12.4 ± 0.9	14.4 ± 1.0
Group 4 DAD + dexamethasone, repeated course administration	0.41 ± 0.08	11.6 ± 0.7	14.8 ± 1.2
Group 5 DAD + betamethasone, single administration	0.50 ± 0.04	13.4 ± 0.4	14.3 ± 0.7

## Data Availability

The original contributions presented in this study are included in the article. Further inquiries can be directed to the corresponding author.
